# Sex differences in the herding styles of working sheepdogs and their handlers

**DOI:** 10.1371/journal.pone.0184072

**Published:** 2017-09-14

**Authors:** Erin Kydd, Paul McGreevy

**Affiliations:** Faculty of Veterinary Science, University of Sydney, Sydney, NSW, Australia; Universidade do Porto Instituto de Biologia Molecular e Celular, PORTUGAL

## Abstract

Working sheepdog trials test the attributes of dogs as well as the dogmanship and stockmanship skills of handlers. They generally include standard elements such as *outrun*, *lift*, *fetch*, *drive*, *shed*, *pen* and *single* to test all facets of the work that dogs perform on a farm. While both male and female handlers participate, these trials are traditionally dominated by male handlers. Both male and female dogs compete on equal terms within the same events. Drawing data from files (n = 60) downloaded from YouTube, the current study explores whether behaviours of dogs and their handlers during sheepdog trials differ between handler gender and dog sex at different levels of competition. It compared the stalking, crouching, chasing and stationary behaviours of dogs in open (n = 28 dogs: 10 females, 18 males) and not-open trials (n = 32 dogs: 20 females, 12 males). The dogs in this study had male (n = 38) and female (n = 22) handlers, whose movement and use of vocal cues and arm elevations were also compared. However, the small sample size and limitations of these videos as a data source should be noted before the results are generalised to the broader field of working-dog behaviour. The results of an REstricted Maximum Likelihood test showed that male handlers spent, on average, significantly more time in the fetch and drive elements than female handlers, but this difference between sexes was present only in not-open events (mean time to Fetch, female handler = 44.07s, male handler = 124.00s, P<0.001, mean time to Drive, female handler = 95.8s, male handler = 152.4, P = 0.010). This may suggest that female handlers of less experienced dogs are better at the early training of these elements. The results showed that male dogs spent more time stationary than female dogs, but only in open competition (male dog predicted mean 6.17s, P = 0.014). Revealing differences between men/women, and between dogs/bitches in this context may identify pairings that complement each other and improve selection, training and handling of working dogs. It is also hoped that ultimately, it will lead to improved welfare for dogs and the livestock with which they interact.

## Introduction

Dogs provide a means of herding and manoeuvring livestock that is quick and relatively easy for livestock producers. Good working sheepdogs are an excellent investment for producers, bringing a high rate of return[1]. Trials for working sheepdogs are designed to mimic the conditions and challenges faced in the day-to-day work of a sheepdog and test the dogmanship and stockmanship skills of the handler. While both male and female handlers participate in sheepdog trials, the sport is traditionally dominated by male handlers, and surveys of sheepdog handlers report more male respondents (69%) than female (31%)[1]. The expansion of the sport into new regions is seeing more women participating with success in competitive sheepdog trials. Furthermore, both bitches and dogs compete within the same classes in sheepdog trials. Despite this, information detailing any differences in technique between gender of handler and the sex of sheepdogs is purely anecdotal. That said, there is evidence of sexual dimorphism in the behavioural attributes of dogs occupying both companion and working roles [2,3].

Working sheepdog trials comprise a number of elements intended to test the skills, training and stamina of the dog. The International Sheep Dog Society (ISDS) runs trials at three levels: nursery for young inexperienced dogs; novice and open for more experienced dogs and handlers. Depending on the level of competition, these trials generally include the standard elements of *outrun*, *lift*, *fetch*, *drive*, *shed*, *pen* and *single* to test all facets of the work that dogs perform on a farm. The trials involve the dogs running out to the flock (*outrun*), moving the sheep (the *lift*) and driving them towards the handler (the *fetch*). Upon reaching the handler, the *drive* is performed, where the flock is moved away from the handler through one or more gates. After the *drive*, a *shed* element may be included, in which sheep are moved to a marked area and selected sheep are removed from the flock by the dog. This is similar to the *single* element, in which a single sheep is removed from the flock. *Penning* involves the dog moving the sheep into a small, penned area and concludes when the handler closes the pen gate.

A number of studies have identified ways in which a human’s gender can influence their interactions with dogs. This may include difference in petting style[4], verbal cues[5,6] or even appearance or body odour, as has been suggested by Wells and Hepper[7].

Physical differences between men and women may also influence the cues they send to dogs. For instance, vocal attributes have been identified as pivotal to success in training of and communication with service dogs[5]. In agility trials, pre-competition testosterone concentrations in male handlers correlate with their dogs’ cortisol concentrations and may act as a predictor of variability in their dogs’ cortisol concentrations during competition[8]. Factors such as these may influence the success of working dog training and affect the outcome of trials. To date, there is no peer-reviewed data on the influence of handler gender on the outcome of sheepdog trials.

In some contexts, despite sexual dimorphism in long bone length and bodyweight, the sex of dogs appears to have no effect on their performance. For instance, the sex of greyhounds is reported to have no effect on speed[9], most probably because females are lighter and so can run as quickly as their male counterparts, despite having shorter long bones. In certain breeds of dog, Serpell and Hsu[10] observed sex differences only in trainability. Despite this, many humans may have a preference for a dog of a particular sex this may be based on a perceived superiority in some desired trait. Differences in boldness and shyness have been recognised to be predictors of performance in working dogs, with bolder animals being the better performers[11]. This mirrors the recently reported opinions of working farm dog handlers, who identified boldness in their dogs as a desirable trait[12]. Although it is possible that bitches and dogs may differ in boldness, there is limited evidence of sex differences in the herding style of working farm dogs.

It is possible that gender differences in innate handler attributes may complement innate dog differences. Optimising the combination of handler and dog has the potential to both improve performances in competitive sheepdog trials and foster more efficient and functional farm work. The quality of the work performed by farm dogs also has implications for animal welfare, since better dogs are expected to minimise the stress imposed on livestock during management practices[13]. The current study aimed to explore whether behaviours of dogs and their handlers during sheepdog trials differ between handler gender and dog sex at different levels of competition. It drew data from files downloaded from YouTube.

## Methods

The behaviour of working sheepdogs and their handlers during competitive sheepdog trials was investigated and scored using videos posted on YouTube. All the data collected was from this site and did not require ethics approval. The trials were generally run in the format of an ISDS sheepdog trial and the dogs used were chiefly Border collies and their crosses. The videos were found using the search terms “sheepdog trials” and “working sheepdogs”. For consistency, only videos with certain features were included. The inclusion criteria were: video footage and audio that were of good quality; only one dog competing at any one time; and the species being herded were sheep. Every video we found that met the selection criteria was included until 30 videos of each handler gender were collected.

Variables, such as gender of handler, sex of dog and level of competition were recorded. Competing animals were described as being dog or bitch, but were not described as being desexed or entire. Level of competition was categorised as either open or not-open (for instance, nursery trials). Open competition is a higher level of competition where competitors can earn points towards championship events. For the current study, competing in open trials was used as an indication of dogs having reached a certain level of proficiency, based on the assumption that the open dogs are more experienced than others. This was chosen as the most appropriate proxy for experience in this study because the exact level of experience of each dog (in terms of trials attended, trials competed, trials won or, more simply, in terms of hours worked) could not be ascertained using this data source. Videos (n = 60) of handlers, male (n = 30) and female (n = 30), were scored. These videos included dogs of both sexes competing in open or not-open level, the numbers of each appear in Table 1.

**Table 1:** 

	Trial–not open		Trial–open	
	Handler gender		Handler gender	
Dog sex	Female	Male	Female	Male
Female	5	15	6	4
Male	5	7	6	12
**Total**	**10**	**22**	**12**	**16**

Distribution of handler gender and dog gender across the videos of open trials (n = 22) and not-open trials (n = 38) scored.

Due to variations in the areas, set-ups and inclusion of various elements within each sheepdog trial, the timing of individual elements was recorded. Typically, trials included the following elements, outlined in Table 2, each of which was timed individually. Some variation in inclusion of different elements was observed for different levels of competition. To the authors’ knowledge, the current report is the first to describe these behaviours operationally.

**Table 2:** Definitions of the commencement and termination of manoeuvres scored in videos of trials.

Element	Definition for timing purposes	
	Start	Finish
Outrun	Dog begins running towards sheep	Dog reaches sheep
Lift	Dog reaches sheep	Sheep begin moving
Fetch	Sheep begin moving	Sheep pass handler and post
Drive	Sheep pass handler and post	Sheep pass through (or near) all gates and reach pen or shedding ring
Shed	Sheep reach stand in shedding ring	Required number of sheep are separated from group
Pen	Sheep are manoeuvred to near the pen	All sheep are in pen and gate closed
Single	Sheep are standing in shedding ring	A single sheep is separated from group

Individual behaviours of handlers and dogs were also recorded (Table 3). The recorded behaviours of handlers were raising an arm/ both arms/ shepherd’s crook, use of auditory cues (whistle or voice) to the dog, and moving or not moving. The recorded behaviours of the dog were moving, not moving, chasing, stalking and crouching.

**Table 3:** Ethogram of dog and handler behaviours recorded, including definitions.

	Behaviour	Definition
Dog	Chase	The dog was running fast (with a galloping gait) around or behind the sheep.
	Stalk	The dog was moving at a slower pace (jog, trot or walk) with head lowered
	Crouch	The dog was stopped and lying (ventrally) flat on the ground facing the sheep
	Moving	The dog was running in a direction away from and not near the sheep (distinct from moving the sheep)
	Not moving	The dog was stationary, either standing or on the ground
Handler	Arm elevation	One or both arms raised away from the handler’s body. This includes raising stick or shepherd’s crook
	Auditory cue	An audio cue, either by voice or whistle, was given to the dog
	Moving	The handler was walking or running
	Not moving	The handler was standing still

Any escape attempt by the sheep was also recorded. Recording the number of occurrences of each of these behaviours within each element was our best effort to adjust for variations in videos such as cut scenes, different course areas and differences in course designs in different levels of competition.

### Statistical analysis

Due to individual differences in each trial, the rate of stalk, crouch and chase events per second (i.e., the mean number of times each event occurred as a ratio of element duration) was measured as well as the average duration of these events. The variables investigated were the level of competition (open or not-open), sex of dog and gender of handler, rate of crouch, stalk and chase by the dog, and arm elevations and vocal cues by the handler.

In this study, half of the handlers were female and half were male. So, while there were not equal numbers of dogs of each gender, the structure of the model was is nevertheless equally proportioned and balanced for the combination of factors of interest. The current investigation focused on any differences between events which were not open and events which were open, the combinations showed imbalance at that level, so the use of a Linear Mixed Model (REstricted Maximum Likelihood or REML) was indicated. This was conducted with a number of steps, first checking the highest order interactions then sequentially until we arrived at main effects. For unbalanced data, we needed to re-run the analysis at each stage once we had excluded a higher-order interaction or (eventually) a main effect (on the basis that it was not significant). We did not perform multiple comparisons, but instead derived the p values from the factorial REML analyses.

Relationships between levels of competition and frequency of crouch, chase and stalk as well as the average duration of stalk, were analysed using a two-sample t-test. The rate of arm movements by the handler and dog sex was also analysed using a two-sample t-test. Relationships between levels of competition, dog sex and time spent not moving were analysed using an REML test, as were handler gender, auditory cue use, rate of arm elevations and competition elements.

## Results

The two sample t-tests revealed several differences in the way dogs in open trials differ in their technique relative to those competing in not-open trials. Open dogs (i.e., those competing in open events) had significantly longer durations of stalk episodes than not-open dogs (open mean stalk duration = 7.772; not-open mean stalk duration = 5.032, t(58) = –3.18, P = 0.002). Open dogs were also less likely to commence a stalk event or chase event than not-open dogs: (mean rate of stalk events per second: open mean = 0.068; not-open mean = 0.094, t(58) = 3.09, P = 0.003) (mean rate of chase event per second: open mean = 0.046, not-open mean = 0.068, t(58) = 2.94, P = 0.005). No significant difference was found between female dogs (mean = 4.94) and male dogs (mean = 3.96) in mean chase duration (t(58) = 1.82, P = 0.074). Open dogs showed a trend towards being less likely to crouch than not-open dogs (mean rate of crouch events per second: open mean = 0.029, not-open mean = 0.043, t(40) = 1.99, P = 0.054). However, this failed to reach statistical significance.

**Fig 1:**
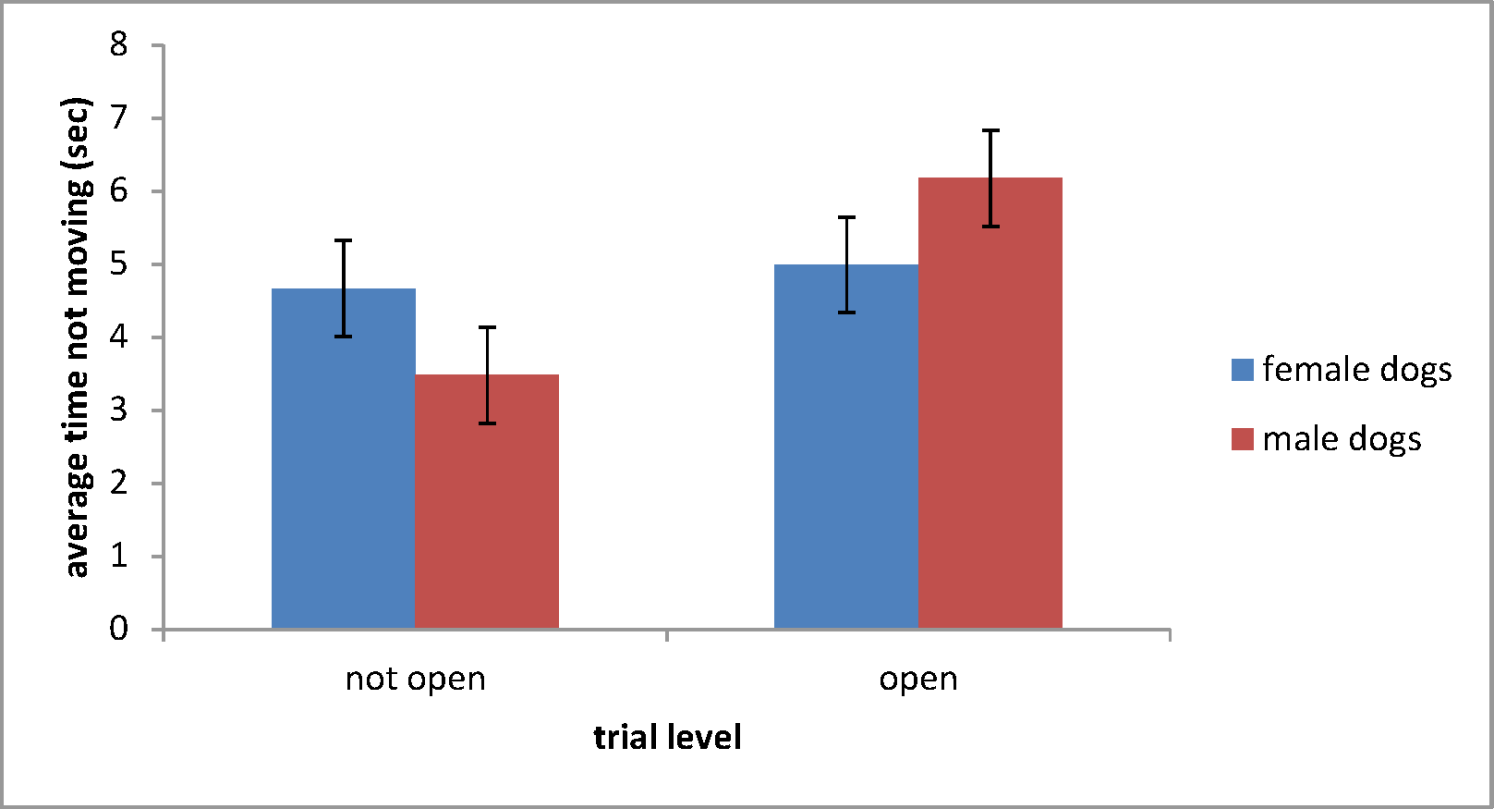
Mean (±SE) time spent not moving by dogs of either sex in not-open and open trials.

Results of an REML test indicated that, in open trials dog sex was related to the time spent not moving (F = 6.45 P = 0.014). Male dogs spent more time immobile than female dogs, but only in open competition (Fig 1).

Overall, there was no significant difference in average duration of the *fetch* and *drive* in open and not-open trials (Fetch P = 0.275 df = 3, Drive P = 0.454 df = 3). However, the results of a REML test showed male handlers spent, on average, significantly more time in the *fetch* and *drive* elements than female handlers, but this difference between gender was only present in not-open events (Table 4, *fetch* P<0.001, *drive* P = 0.010). For not-open trials, there was no significant difference observed between handler gender (Fetch P = 0.534, Drive P = 0.433).

**Table 4:** 

Element	Competition level	Female handlers	Male handlers	SE of difference	P value
Fetch	not open	44.07	124.00	17.47	<0.001
	Open	101.42	89.56	18.92	0.534
Drive	not open	95.8	152.4	21.3	0.010
	Open	128.7	110.2	23.4	0.433

Mean time (sec) spent in elements of a trial where a significant difference was observed between handlers of either gender.

**Fig 2:**
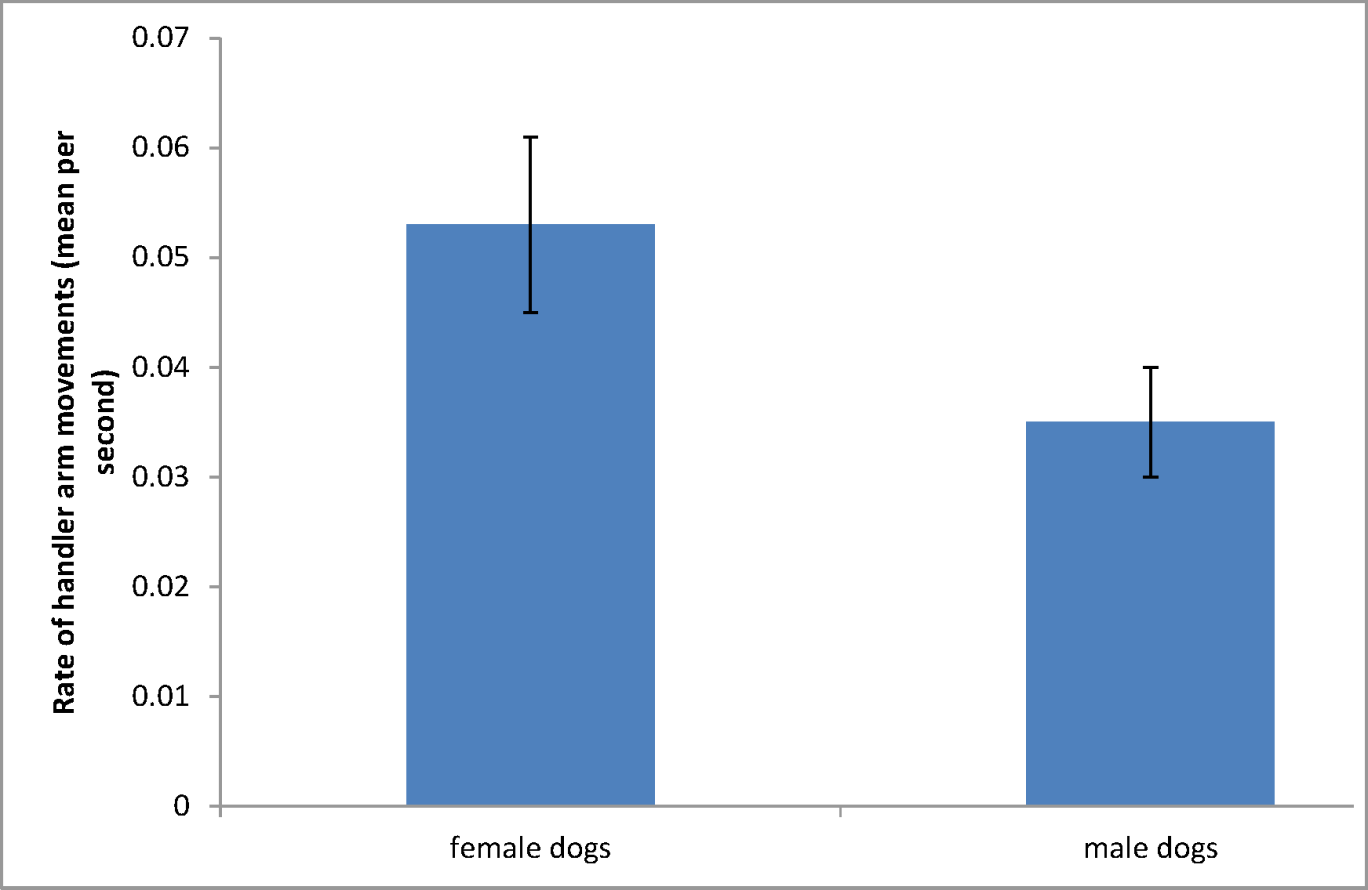
Mean (±SE) rate of arm elevations made by handlers per second when dealing with dogs of different sexes.

In the results of a two-sample t-test, a difference was observed in the rate at which handlers raised their arms when dealing with dogs of either sex. Handlers of both genders raised their arms more frequently when dealing with female dogs (mean = 0.053 arm movements per second) than when dealing with males (mean = 0.035 arm movements per second) (Fig 2, t(58) = 2.09, P = 0.041).

The results of an REML test showed that the rate of auditory cues given had no significant association with either dog sex or handler gender, nor was there any interaction or effect in any element of the trial. Likewise, a bivariate REML output indicated that handlers who vocalise more frequently were not found to have any significant interaction with frequent arm-raising.

## Discussion

The current data suggests that both dog sex and handler gender appear to have some influence on the style of approach in competitive sheepdog trials, and the way dogs and handlers interact with each other. However, the differences observed between males and females appear not to be uniform and vary with different elements of stock work, as well as with experience.

### Dogs

The results of the current study confirm what has been shown by a number of previous studies–that male and female dogs behave differently. In the literature, sex differences in behaviour are reported to be highly context-specific and also breed-specific [10,11]. The current study shows that the sex of Border collies competing in sheepdog trials influences their herding style. Interestingly, the observed differences in not moving between male and female dogs are revealed only in the dogs competing in open trials.

By differentiating between dogs competing in open trials from those in not-open trials, we hoped to expose the behavioural attributes of the more-advanced dogs. This was indeed the case, with evidence that dogs competing at different levels of competition behave differently. These differences were observed in the duration and frequency of stalk events as well as the frequency of chase and crouch events, with open dogs performing all these events less frequently. The sex differences observed were also evident in dogs competing in open trials. This may reflect the level of experience and arousal of the dogs.

Compared with open dogs, novice dogs may be younger, less focused and over-stimulated by the atmosphere associated with trials. Being less experienced and having accumulated less training are also likely to lead to sub-optimal movement of the sheep, and may explain under-developed skills in reading and predicting sheep behaviours and reacting appropriately. This could potentially account for the increased prevalence of herding behaviours, such as crouching and chasing, observed in the not-open competitions. These findings suggest that many of the not-open dogs are still learning the finer points of their job and, as such, may be trialling different behaviours to control the movement of the sheep. Further studies on trial scores could focus on the interactions of age, experience and sex.

Importantly, this study found that male dogs competing in open trials, were more likely to remain stationary (not moving) than female dogs. Taken in the light of differences in stalking reported here between open and not-open dogs, it may be that stalking and being stationary are intimately entwined. As steady, controlled movements of the sheep are considered indicators of a good herding dog[14], it can be inferred that male dogs, in general, are better at this particular aspect of sheepdog trials. Whether this steadiness correlates with higher scores and more accuracy when moving stock through the gates, and whether females excel in other aspects of herding work are areas for future study.

### Handlers

Handlers of both genders behaved differently with dogs of either sex. Male handlers spent, on average, significantly more time in the fetch and drive elements than female handlers, but only in not-open events. This may suggest that the female handlers of less experienced dogs are better at the early training of these elements but it is far from clear why or how. Both men and women raised their arms more when dealing with bitches. This suggests that they are aware of and responsive to the differences in style between dogs of each sex (as discussed above). However, in many instances in this study the rate of auditory cues from handlers of both genders towards dogs of either sex showed no observable differences.

The absence of any correlation between arm cues and auditory cues suggests that handlers use these discriminative stimuli differently and in different contexts within trials. It may be that arm movements are used primarily to guide the sheep and are less often used as cues for the dogs, whereas auditory cues are likely to be intended exclusively for the dog. That said, if trainers are consistent in the array of signals used, dogs are likely to find them easier to read[15]. It has also been noted that Border collies perform exceptionally well in tasks that depend on human pointing[16] and so, perhaps, along with other breeds, do not require repeated arm movements from handlers to detect the discriminative stimulus. It is also worth emphasising that verbal cues may bear repetition because, in contrast to visual cues, they are subject to environmental influences, such as wind direction, crowd noises and distance that may inhibit the dog’s ability to receive them.

Previous studies have observed that female handlers, when communicating with dogs, use verbal communication similar to that used when communicating with infants, sometimes called *motherese*[6], but frequent use of whistles in addition to vocal cues in sheepdog trials largely prevents this from occurring in competition contexts. So, a difference in frequency of auditory cues between handler genders may have been expected, but none was revealed.

There were some limitations to the current study that should be noted, before the results are generalised to the broader field of working-dog behaviour. Foremost among these are the drawbacks imposed by using publicly available video footage from YouTube. While this resource provides many benefits to the canine research community, it is subject to editing by the original poster, who may have included only highlights of the dog’s behaviour in the trial or may have cut certain elements of the performance. The videos included some individual notable differences such as in the layout of trials, different terrain, shape and size of the trial paddock, video and audio quality and editing choices that may include cut scenes and close-up footage. Furthermore, there is limited availability of videos that fit the criteria for inclusion into this study, leading to a restricted sample size. For these reasons, we recommend caution in interpreting the results to limit the influence of such unseen editorial effects.

### Conclusion

Dogs use individual styles when herding sheep during sheepdog trials, and this may be partly explained by the sex of the dog. While no obvious distinctions between handlers of different genders competing in sheepdog trials were observed in this study, there were indications that all handlers were able to adjust their style to suit the different herding styles of dogs of either sex. Findings such as these can improve selection, training and handling of working dogs and ultimately lead to improved welfare for the dogs and livestock they interact with.

## Supporting information

S1 File Dataset. (XLSX)
